# Influencing recommendation algorithms to reduce the spread of unreliable news by encouraging humans to fact-check articles, in a field experiment

**DOI:** 10.1038/s41598-023-38277-5

**Published:** 2023-07-20

**Authors:** J. Nathan Matias

**Affiliations:** 1 Department of Communication, Cornell University, Ithaca, NY USA; 2 Center for Advanced Study in the Behavioral Sciences, Stanford University, Stanford, CA USA

**Keywords:** Human behaviour, Computational science

## Abstract

Society often relies on social algorithms that adapt to human behavior. Yet scientists struggle to generalize the combined behavior of mutually-adapting humans and algorithms. This scientific challenge is a governance problem when algorithms amplify human responses to falsehoods. Could attempts to influence humans have second-order effects on algorithms? Using a large-scale field experiment, I test if influencing readers to fact-check unreliable sources causes news aggregation algorithms to promote or lessen the visibility of those sources. Interventions encouraged readers to fact-check articles or fact-check and provide votes to the algorithm. Across 1104 discussions, these encouragements increased human fact-checking and reduced vote scores on average. The fact-checking condition also caused the algorithm to reduce the promotion of articles over time by as much as −25 rank positions on average, enough to remove an article from the front page. Overall, this study offers a path for the science of human-algorithm behavior by experimentally demonstrating how influencing collective human behavior can also influence algorithm behavior.

## Introduction

In recent years, communications technologies have broadened access to so much information that society relies on automated systems to filter, rank, suggest, and inform human thoughts and actions. These algorithms have been implicated in many complex patterns in collective human behavior, including misinformation^1^, voting behavior^2,3^, social movements^4,5^, charitable donations^6^, sexist and racist harassment^7,8^, public safety^9^, and extremism^10^. Consequently, the work of maintaining democratic societies now involves managing algorithms as well as people^11–13^. Explaining collective human and algorithm behavior has become an urgent scientific question due to these pragmatic concerns^14^.

Consider how humans and algorithms influence each other to spread false information. When people post news articles to online platforms such as Reddit, social algorithms rank the articles and display them to readers in a news feed^7^. As people read, click, comment, and vote to promote or demote the article, the news aggregator algorithm observes their actions and adapts its own behavior in real-time, updating the prominence of an article in relation to the supply of available content^15–17^. When a false article is ranked more highly, more people see and respond to it. The algorithm’s behavior then further adapts, potentially showing the article to yet more people. These adaptations occur in parallel to regular software changes that engineers make in response to adversarial attempts to manipulate the algorithm^18,19^.

Although many have identified human-algorithm feedback as an important problem, both computer scientists and social scientists report lacking reliable enough explanations of this dynamic to govern the spread of false information or other harmful content^14^. Many social scientists studying human–computer interaction have studied the influence of algorithms and software design on individual human behavior^20^. For example, when researchers evaluate the experience design of fact-checking interventions, they are studying individual beliefs and sharing behaviors as they are shaped by psychology, organizations, social structures, and culture^21–28^. Similarly, when researchers study how aggregated rankings influence human herding, they are showing how user experience design influences individuals toward patterns of collective behavior^29^. On one website that displayed ratings to readers, a single positive rating from researchers caused herding, increasing final ratings of content by 25% on average^30^.

Despite significant progress on theorizing the influence of controlled software activity on human behavior, computer scientists and technology firms have struggled to develop general theories about the influence of human behavior on algorithms^13,14^. In the absence of general theories of human-algorithm feedback, one technology firm alone spends over two billion dollars annually to surveil and censor human behavior, protecting algorithms from potentially-harmful human influence^31–33^. How could this situation arise when computer software can reliably implement precise instructions? While engineers directly create the mechanisms of social algorithms, computer scientists are also ambivalent about their ability to predict human-algorithm behavior in the field^34^. In controlled, lab-like circumstances, algorithms are highly predictable^35–38^. To date, most efforts to understand algorithm behavior have worked under these lab conditions, opening the “black box” of data and software code to audit an algorithm in the lab^39–41^. But when social algorithms adapt to human behavior in the “rich thicket of reality”^42^, both technologists and social scientists argue that they lack empirically-grounded theory to reliably predict and change human-algorithm feedback^14,31,43^. Because social algorithms adapt to diverse, changing social contexts and undergo regular changes from engineers, some scientists even argue that generalizable knowledge about algorithm behavior could be impossible to obtain^44^.

A complete explanation of human-algorithm feedback would need to account for at least four steps in a cycle of mutual influence. When designing systems, computer scientists define and weight the measurements of collective human behavior that the algorithm will attend to^15^. Even when these mechanisms are trade secrets, scientists and regulators still need to understand their workings. In response, a growing field in computer science on fairness, accountability, and transparency now investigates the behavior of algorithms without access to knowledge about their internal mechanisms^39,45^. Scientists have also made progress on the next two parts of the feedback loop. Interdisciplinary scholarship in human–computer interaction and the social sciences study the influence of algorithm-generated stimuli on individual behavior^29,46^. Cybersecurity researchers study fraudulent attacks from non-human accounts that can artificially-influence algorithm behavior. These computer scientists have developed language to describe hypotheses for how algorithms interact with accounts that act in non-human ways^37,38^. Overall, while engineers can sometimes describe the workings of algorithms and social scientists can explain how those algorithms influence the mechanisms of human behavior, the influence of humans on algorithms has puzzled both technologists and social scientists alike^14^.

To fully account for human-algorithm feedback, scientists need theories that explain the influence of human behavior on the social algorithms that adapt to humans. Consider the case of crowdsourced fact-checking. Laypeople’s judgments about the trustworthiness of sources correlate strongly with those of professional fact-checkers and simple interventions can reduce people’s sharing of misinformation^47,48^. Yet encouraging human fact-checking might also change algorithm behavior: if fact-checkers interact more with suspect information, news aggregation algorithms might interpret that behavior as evidence of greater popularity. If aggregators respond to this popularity by promoting those articles more widely, the second-order effect of fact-checking could increase rather than reduce collective misunderstanding. Whether effects on algorithm behavior are consistent across all systems or are just a localized effect until the next software change, both policymakers and scientists would benefit from knowing that such effects could occur and estimating those effects.

**Figure 1:**
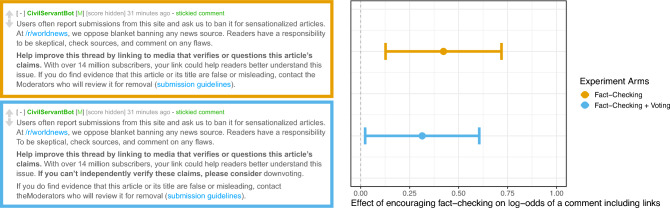
In a logistic regression, encouraging fact-checking increased the chance that individual comments would include links to further evidence (n = 35,090 comments in 869 discussions) (p for Fact-Check = 0.003, p for Fact-Check + Voting = 0.032) (Table S1).

If human behavior influences social algorithms, can we influence algorithm behavior by influencing humans? Using a large-scale field experiment, I estimate the effect of encouraging human fact-checking on the subsequent behavior of an influential news algorithm. To conduct this experiment, a software program^49^ identified recently-submitted articles in r/worldnews, a discussion community of over 14 million subscribers on the Reddit platform. This community routinely shares, comments, and votes on news articles about places outside the United States. Focusing on news websites that the community considered regular publishers of inaccurate claims, the software observed when someone submitted a link for discussion and randomly assigned the discussion to receive one of three conditions. In the first condition, readers were shown a persistent message encouraging them to fact-check the article by commenting with links to further evidence (Fig. 1). The second condition added a sentence encouraging readers to also consider down-voting the article to potentially reduce its position in the rankings (Fig. 1). In the control group, no action was taken. Within discussions, I observed whether reader comments included links to further information. Every four minutes, software observed an article’s aggregate vote score from readers and recorded the rank position that the community’s news aggregator algorithm gave the article.

## Results

Among humans, both fact-checking conditions increased the chance that an individual comment would include links to evidence in discussions of articles from frequently inaccurate news sources (Fig. 1, Table S1, p = 0.003 and p = 0.032). Since these nudges successfully influenced human commenters activity, I was able to estimate the second-order effect on the behavior of the news aggregator.

Because I expected that encouraging fact-checking would increase commenting activity, I predicted in the experiment pre-registration that encouraging fact-checking would indirectly influence the algorithm to increase the rank position of unreliable news articles, growing their visibility. Encouraging voting alongside fact-checking, I predicted, might dampen that effect^50^. The pre-registered analysis failed to distinguish any second-order effect (Table S4, p = 0.079, 0.187), likely because the model did not account for interference in cases of multiple treated articles appearing in the same rankings.

**Figure 2:**
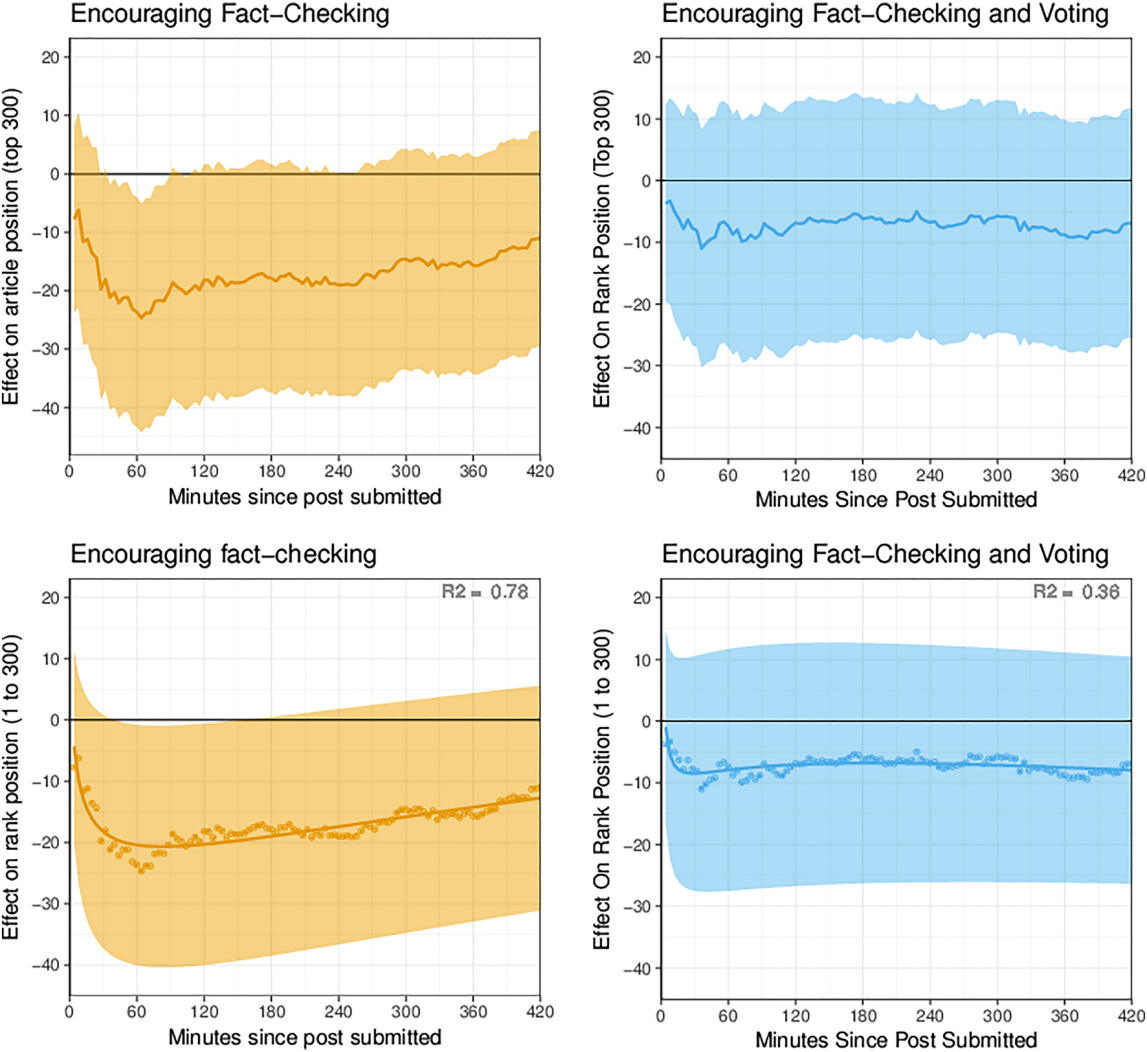
While encouraging fact-checking reduces the rank position of an unreliable news article (left), encouraging readers to fact-check and vote has no discernable effect on rank position (right). The first row shows the effect size and adjusted 95% confidence intervals for 105 linear regressions estimating the effect on rank position at a moment in time. The second row shows the fitted effect sizes and fitted, adjusted confidence intervals estimated by a cubic polynomial model (Table S5).

In an exploratory longitudinal analysis that accounted for this interference, I found that encouraging fact-checking caused Reddit’s algorithm to make unreliable news less visible. In a series of linear models estimating the average treatment effect at four minute intervals (Table S6), encouraging fact-checking reduced the algorithmic ranking of unreliable news articles over time by as many as -25 rank positions out of 300 on average, at the moment of the greatest effect (Fig. 2). On Reddit, where the first page of results on mobile and desktop devices tends to include fewer than ten articles, an effect of this size would prevent many readers from ever seeing the article. While I predicted that the encouragement to downvote would reduce an article’s rank position compared to both the control group and fact-checking conditions, I failed to find evidence of such a reduction. The average estimated effect on article rankings in the voting condition was never lower than the fact-checking condition, and any differences between the voting condition and the other experiment arms are indistinguishable from chance in this sample (Fig. 2).

**Figure 3:**
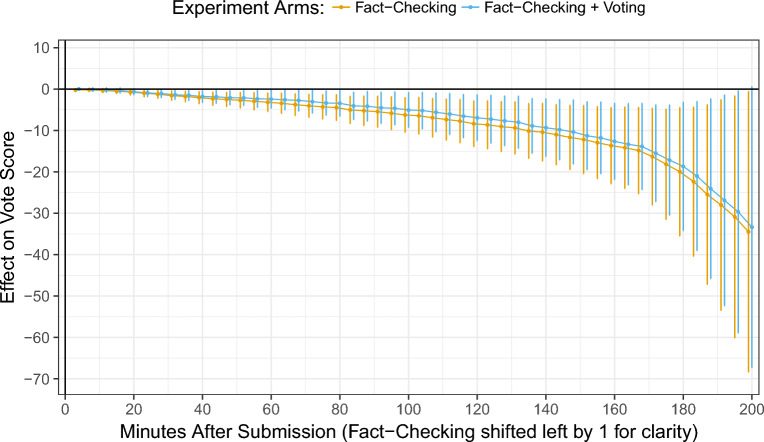
Both interventions reduced the vote score of the treatment groups compared to the control group. This chart shows the effect size and 95% confidence intervals for 50 linear regressions estimating the effect on vote score at a moment in time in the first 200 min after the article was posted.

What mechanism explain this second-order effect on algorithm behavior? In a follow-up exploratory analysis, I found that encouraging fact-checking and fact-checking + voting caused an article to receive a lower aggregate vote score over time compared to articles that did not receive the encouragement (Table S7, Fig. 3).

The effect of influencing algorithms by influencing humans can also be predicted over time. Since news aggregators are designed to reward novelty, articles begin at a similar position in aggregator rankings, sometimes rise to prominence, and then recede until they are ranked too low to observe^15,16^. Consequently, any effect on the ranking of a news article over time will start small, grow to the point of greatest variance between articles, and then recede. The estimated effects on rankings at a moment of time can be modeled with a cubic polynomial curve over the log-transformed age of the article discussion (Table S5). In the early minutes after a news article is submitted, the average treatment effect is small enough to be unobservable in this sample. The average reduction in rank position quickly grows in the first sixty minutes, reaches a maximum effect size shortly after and then declines gradually over time (Fig. 2).

## Discussion

As expected, readers do engage in fact-checking behaviors when asked. In the worldnews discussion group, which received 494 comments per day on links to routinely-inaccurate news sources, encouraging fact-checking could cause 27 more comments per day to include at least one link, on average. While this study does not evaluate the information quality of those links, these findings offer promising evidence on reader participation in fact-checking.

The second-order effects on algorithm behavior were more surprising. The community and I predicted that encouraging fact-checking could increase commenting activity on articles, causing Reddit’s news aggregator to further promote news from often-inaccurate sources. Instead, the intervention caused articles from those sources to receive lower rankings from the aggregator over time compared to the control group, enough to move an article headline out of view for most readers.

While a single experiment cannot explain all of the mechanisms of psychology, social structure, and algorithm design in the full cycle of this feedback loop, the findings do partially explain how the intervention influenced Reddit’s news aggregator. In a public description of their news algorithm and in source code that the company obfuscated to resist manipulation, the company emphasized the importance of scores that aggregated upvotes and downvotes^51,52^. Since both interventions caused submissions to receive a lower aggregate vote score over time, the intervention’s effect on voting was likely one mechanism of influence on algorithm behavior, a finding consistent with the published source code (Table S7, Fig. 3). If the algorithm attended to comment counts, any effects on the number of comments might have also influenced the news aggregator. Discussions in the condition encouraging fact-checking had a mean number of 12 comments, compared to 61 in the control group and 22 in the condition that encouraged voting (Table S2). Yet differences in comment counts might only be correlated with the effects on rankings. Fact-checked articles, if ranked lower, would likely have received less attention and fewer comments.

This finding has several limitations. First, since treatment and control articles sometimes appeared in the same ranking, reductions in the rank position of treatment articles may also have increased the rank position of control group articles, even though the models adjust for this spillover effect. Second, because effects on rankings are dependent on the supply of content, the magnitude and direction of any effects in other circumstances may be different. Third, since I was unable to observe individual vote decisions and had no access to internal states of the algorithm, these findings leave many questions remaining about the mechanisms behind the observed effects. Furthermore, because news aggregators vary widely and because their designs change over time, the results from one experiment cannot provide general guidance on second-order effects on other algorithms. Finally, this study contributes evidence into an adversarial arena of misinformation and its responses. Like any experiment that does not test variations of adversarial behavior, this study cannot provide guidance on the reliability of the observed effect in the presence of competing actors aware of this study’s findings.

In this study, I have tested and explained an important link in a cycle of causality between human and algorithm behavior. This study demonstrates how influencing human behavior can also influence social algorithms to behave differently downstream. Behavioral interventions such as encouraging fact-checking can involve the public in evaluating information quality. These interventions also cause second-order effects on algorithms that influence human beliefs and behaviors in turn. In the time since I conducted this study, the need for causal inference on human and algorithm interactions became even more urgent when global platforms with billions of users including Facebook, YouTube, and Twitter developed information quality algorithms that directly rely on public guidance^53–55^.

Because this study tests a novel hypothesis in a changing environment, contributions to theory can also be found in the questions this study raises. First, questions about the mechanisms that drive these second-order effects represent theoretical opportunities for the study of human and machine behavior. Second, scientific questions about the behavior of adaptive algorithms represent important challenges for the generalizability of social research. As adaptive social algorithms play a greater role in social behavior, scientists will need to advance our capacity to develop generalizable knowledge about these second-order effects^44^. Since human-algorithm feedback can exert substantial power in society, these questions for social and computing theory also have urgent, pragmatic importance to society.

## Methods

### Hypotheses in psychology, computer science, and community science

By testing a hypothesis about human and algorithm behavior, this study engages with three different notions of hypotheses from social psychology, computer science, and community science. Psychologists have described recommender systems as software inspired by psychological processes of reinforcement learning^56^. To these social scientists, experiments set out to validate theory about cause, effect, and underlying processes that are incompletely understood^57^. Computer scientists might frame the experiment differently, as a security attack that relies on low or high information about the underlying software design. These researchers study influences on algorithm behavior as threats from coordinated non-human actors that do not behave like humans, in an arms race between algorithm designers and adversaries with varying levels of prior information. In this model, a successful attack does not reveal a scientific discovery about the algorithm or even human-algorithm behavior but a repairable failure in defenses against non-human behavior^37,38^. Finally, traditions in community science and action research derive testable scientific theory from the intuitions of people with direct experience of the phenomenon^58–60^.

The study’s pre-registered hypothesis is that influencing humans to contribute greater numbers of comments and votes would provide positive reinforcement to the algorithm, causing it to rank an article more highly on average. Cybersecurity researchers would recognize this as a low-information hypothesis, since the company had published avowedly incomplete, outdated^52^ source code that outlined the basic structure of the default “Hot” algorithm^51^. The 12 lines of incomplete source code claim that the algorithm ranked posts based the difference in upvotes and downvotes, as well as the number of seconds since a post was made (the source code also includes “Top” and “Controversial” algorithm options that were not active by default)^51^. As community science, this hypothesis was also developed in hours of video and text conversations with dozens of community leaders over several months. Based on their experience, community members expected that comments might also be important inputs for the algorithm, either directly, or indirectly through the herding effect from readers interpreting higher comment counts as more popular discussions. Finally, psychologists would recognize the hypothesis as a test of a theory in the absence of complete knowledge of the underlying mechanisms. The full hypothesis integrates the theory-validating goals of psychology, the partial technical information of computer science, and the participant expertise of community science.

### Experiment procedure

To conduct this experiment, I worked with volunteer moderators of the r/worldnews community on the Reddit platform, a group that shares, comments, and votes on news articles about places other than the United States. When the experiment began, this English language community had over 14 million members. In a 6-week period from mid-September 2016 into October, 914 articles per day were submitted by the community on average, 2.4% of which were from news sites that readers frequently report to moderators for being inaccurate, unreliably sourced, and overly sensational in their claims. Moderators compiled a list of these publishers to include in the experiment. Selected domains included dailymail.co.uk, express.co.uk, mirror.co.uk, news.com.au, nypost.com, thesun.co.uk, dailystar.co.uk, and metro.co.uk. Of these articles, 46% were permitted by moderators. Since several hours can elapse before moderators remove an article, even these articles were viewed and discussed by readers. While community members regularly reported unreliable sources to moderators, the community did not maintain a parallel list of preferred sources.

I conducted the field experiment with CivilServant, software that accessed the platform through an application programming interface (API)^49^. The study was conducted in compliance with relevant guidelines and regulations, in a protocol approved by the Massachusetts Institute of Technology Committee on Use of Humans as Experimental Subjects, which waived informed consent. For this reason, I also sought and received community consent for this study. Moderators granted CivilServant moderator-level access, allowing it to collect near real-time information on community activity and automatically post the experiment interventions. The software queried Reddit for information about news articles every sixty seconds and information about their ranking position every four minutes. Articles were included in the experiment if the news link was from a website domain that moderators considered a frequently-unreliable source. In the control condition, the software took no action. In two treatment conditions, the software posted persistent announcements to the top of conversations. These messages were displayed beneath the article headline as the top-most comment to anyone reading the discussion. In the fact-checking condition, the message encouraged readers to research and share links to further evidence about the story being discussed (Fig. 1). In the fact-checking and voting condition, the message also included a sentence encouraging readers to directly influence the ranking algorithm by clicking a “downvote” button on low-quality news articles (Fig. 1). Experiment conditions were block-randomized by time into groups of 12, applying each arm four times in each block. Blocks where any software error prevented full data collection were removed.

Both fact-checking interventions encouraged an un-structured, deliberative group process. Participants were encouraged to review the article’s claims, research further information, and contribute to a public conversation in the primary space for discussing the article. This group process differs from other forms of volunteer fact-checking that focus on individual claims and aggregate private, individual ratings from people who do not have the opportunity to deliberate^47,61^.

I began the study on November 27, 2016. Since the Reddit platform changed the design of their ranking algorithms and input metrics early in the study^52^, this analysis includes 1,104 news discussions from December 7, 2016 to February 15, 2017. The experiment software made observations of news articles, comments in discussions about those articles, and the rank position of articles over time.

Participants made 35,090 experiment-eligible comments of any kind in 869 news discussions among the 1104 discussions included in the experiment (Table S2). In the analysis, I omit experiment intervention comments and 345 comments made by five common automated systems (autotldr, Mentioned_Videos, DailMail_Bot, youtubefactsbot, HelperBot_). Within eligible comments, I observed a binary value of whether a comment included at least one link to further evidence. Within these comments, 2773 linked only to other Reddit discussions or to image-hosting website subdomains (Reddit, img, image, giphy, quickmeme). I did not label these domains as links to further evidence.

For each news article submitted, the software observed the time that the discussion was started. Moderators sometimes remove articles from discussion for being duplicates or violating community policies. Moderators also sometimes reinstate removed articles if their original decision was mistaken. The system recorded a binary adjustment variable indicating if the article discussion had been conclusively removed at the conclusion of the experiment (Fig. S1).

To observe second-order effects on the ranking behavior of the Reddit news aggregator, the experiment software took samples every four minutes from the top 300 recommendations made by the worldnews community’s primary aggregator, the “hot” ranking on Reddit. The software was able to observe the top 300 ranking items for 516 articles from January 13, 2017 to the end of the experiment. I used ranking observations of the top 300 ranked items to create a longitudinal dataset of the rank position of every news article for the seven hour period after it was first opened for discussion (Table S3). The software observed the ranking position of each news link every four minutes for seven hours, a total of 105 samples per article. The measure of rank position ranges from 0 to 300. A value of zero indicates that the news article did not appear in the top 300 during that observation. A value of 1 indicates the least prominent rank position, and a value 300 indicates the most prominent position (Fig. S1). When moderators remove an article, it is also removed from the rankings and receives a measure of zero in the longitudinal dataset for every subsequent snapshot unless reinstated. Data on top 300 rankings is available for 516 articles in a subset of complete randomization blocks.

### Analysis

I conducted two analyses testing the effect on human and algorithm behavior as specified in the pre-analysis plan, adjusting them using the Bonferroni method for two comparisons^50^. I also fit an exploratory post-hoc longitudinal analysis of rank position over time, adjusting it for three comparisons.

In the first analysis, a logistic regression, I estimated the average treatment effect on the chance of a comment including links to evidence. Since the experiment randomized on discussions rather than people, I adjusted standard errors using the Huber-White method for comments clustered within discussions (Fig. 1, Table S1)^62,63^. To test hypotheses about average treatment effects on rank position, the pre-analysis plan specified a linear regression model predicting differences in the maximum rank position achieved by a news article in the seven hours after it was posted for discussion. In this model, I failed to reject the null hypothesis of a difference in maximum ranking between any treatment and the control group on average (Fact-Check p = 0.088, FactCheck + Voting p = 0.193) (Table S4).

Since the model for maximum ranking estimates the average effect on a single measure of many rankings over time, this imprecision may result from an inability to account for interference from other treated articles that also appeared in the rankings. To account for this interference, I conducted a post-hoc longitudinal analysis of the effect on the rankings at a moment in time.

The longitudinal analysis estimates the effect of the interventions on the relative aggregator ranking of a news article at a period in time. In this analysis, I fit 105 linear regression models, one model for each four-minute sample in the first 7 hours after an article was submitted. These models estimate the average treatment effect on rank position at a certain elapsed time for that article, without making assumptions about the shape of the curve taken by an article through the rankings. A similar series of linear regressions was previously employed in randomized trials estimating human reactions to recommender system behavior^64^.

Each model in the longitudinal analysis estimates the average treatment effect of the two interventions (*β*_1_*TA* and *β*_2_*TB*) on the rank position of an article at a given time after submission (*Position*_*t*_), compared to the control group. I specified the model as follows:

$$Position_{t} = \alpha + \beta_{{1}} TA + \beta_{{2}} TB + \beta_{{3}} P_{t} + \beta_{{4}} SA_{t} + \beta_{{5}} SB_{t} +\epsilon.$$

The models of rank position adjust for whether the article was permitted or had been removed at that time (*P*_*t*_). The model also includes variables used to adjust for spillover: the number of other news articles in the top 300 that received either treatment (*SA*_*t*_ and *SB*_*t*_) (summary statistics are in Table S3).

Table S6 includes coefficients and test statistics for each of the 105 linear regression models illustrated in Fig. 2. These results are the basis for the cubic polynomial model described in Table S5 and Fig. 2.

### Estimating the effect on vote scores

To investigate questions related to the mechanism behind the observed effect on rank position, I estimated the effect of the interventions on the vote score of a news article at a period in time. In this analysis, I fit 105 linear regression models, one model for each four-minute sample in the first seven hours after an article was submitted. Each model estimates the average treatment effect of the two interventions (*β*_1_*TA* and *β*_2_*TB*) on the vote score of an article at a moment in time (*Score*_*t*_), compared to the control group. I specified the model as follows:

$$Score_{t} = \alpha + \beta_{{1}} TA + \beta_{{2}} TB + \beta_{{3}} P_{t} + \beta_{{4}} SA_{t} + \beta_{{5}} SB_{t} +\epsilon.$$

The models of rank position adjust for whether the article was permitted or had been removed at that time (*P*_*t*_). The model also includes variables used to adjust for spillover effects: the number of other news articles in the top 300 that received either treatment (*SA*_*t*_ and *SB*_*t*_) (Summary statistics are in Table S3). A plot of the estimated effects with 95% confidence intervals is available in Fig. 3, and full estimates are available in Tables S7.

## Supplementary Information

Supplementary Information.

## Data Availability

Anonymized datasets analyzed from this study are openly available on the Open Science Framework https://osf.io/m98b6/.
