# Supplementary material for: Influencing recommendation algorithms to reduce the spread of unreliable news by encouraging humans to fact-check articles, in a field experiment
Source: Sci Rep. 2023 Jul 20;13:11715. doi: 10.1038/s41598-023-38277-5 (PMC10359256; doi:10.1038/s41598-023-38277-5)
Supplement: Supplementary file 1 — Supplementary Information. [file 41598_2023_38277_MOESM1_ESM.docx]

# Supplementary materials

Supplementary Text Figure S1

Tables S1 to S7

Without Adjustment Main Model

Intercept *−*3*.*284*∗∗∗ −*3*.*257*∗∗∗*

(0*.*122) (0*.*103)

Encourage Fact-Checking 0*.*435*∗∗* 0*.*423*∗∗* (0*.*146) (0*.*132)

Encourage Fact-Checking + Voting 0*.*320*∗* 0*.*314*∗*

(0*.*139) (0*.*130)

| Article Permitted |  | *−*0*.*032  (0*.*100) |
| --- | --- | --- |
| Num. obs. | 35090 | 35090 |
| Pseudo R2 | 0*.*005 | 0*.*005 |
| L.R. | 46*.*415 | 46*.*679 |

*∗∗∗p <* 0*.*001, *∗∗p <* 0*.*01, *∗p <* 0*.*05 adjusted for 2 comparisons. Stderrs clustered on discussions.

Table S1: Encouraging fact-checking in an online discussion of news from regularly-inaccurate sources increased the chance that a comment would include links to further evidence.

|  | Control | Fact-Checking  (A) | Fact-Checking  and Voting (B) | Total |
| --- | --- | --- | --- | --- |
| Articles with comments | 291 | 300 | 278 | 869 |
| Articles permitted | 47% | 44% | 42% | 44% |
| Comments | 22,286 | 4,550 | 8,254 | 35,090 |
| Mean Comments per article | 60.6 | 12.4 | 22.4 | 31.8 |
| Comments with links | 805 | 249 | 405 | 1,459 |

Table S2: Summary statistics for comments in discussions of articles from regularly- inaccurate publishers.

300

200

100

0

300

Ranking Position

200

100

0

300

Article Permitted

False True

200

100

0

0 1 2 3

4 5 6

7 0 1

2 3 4 5 6 7

Hours Since Article Submitted

Figure S1: Rank position over time for six example articles from regularly-inaccurate news publishers. A rank position of 0 indicates that the news article was not featured in the 300 articles recommended by Reddit’s aggregator at that time. Observed ranks range from 1 to 300, with 300 as the most prominent. When an article is removed by moderators, it is removed from the rankings and receives a value of 0 for the remaining time.

|  | Observed Top 300  Ranked Articles | | |
| --- | --- | --- | --- |
| Start Date  End Date Article Count | Jan 13, 2017  Feb 15, 2017  516 | | |
|  | Min | Mean | Max |
| Treatment articles in top N (A)  Treatment articles in top N (B) | 0  0 | 1.7  2 | 6  7 |

Table S3: Regression adjustment variables for models estimating effects on the rank position of a news article over time

Without Adjustment Main Model

Intercept 27*.*64*∗∗∗* 30*.*75*∗∗∗* (1*.*56) (3*.*21)

Fact-Checking *−*4*.*55 *−*4*.*45

(2*.*21) (2*.*21)

Fact-Checking + Voting *−*3*.*70 *−*3*.*67

(2*.*21) (2*.*20)

Article Permitted *−*3*.*63*∗*

(1*.*82)

Hour Posted *−*0*.*24

(0*.*53)

Hour Posted 2 0*.*00

(0*.*02)

Weekend 3*.*29

(2*.*12)

R2 0.00 0.01

Adj. R2 0.00 0.01

Num. obs. 1104 1104

RMSE 29.94 29.89

*∗∗∗p <* 0*.*001, *∗∗p <* 0*.*01, *∗p <* 0*.*05 adjusted for 2 comparisons

Table S4: A linear regression estimating the average treatment effect on the maximum seven-hour rank position of articles from frequently-inaccurate publishers fails to observe an effect from encouraging fact-checking or fact-checking and voting, after adjusting for multiple comparisons.

Fact-Check Effect in Top 300 Intercept 10*.*785

(6*.*637)

ln Minutes 9*.*340

*−*

(5*.*307)

ln Minutes2 0*.*566

*−*

(1*.*338)

ln Minutes3 0*.*240*∗* (0*.*107)

R2 0.780

Adj. R2 0.774

Num. obs. 105

RMSE 1.507

*∗∗∗p <* 0*.*001, *∗∗p <* 0*.*01, *∗p <* 0*.*05

Table S5: A cubic polynomial model of average treatment effects from encouraging fact- checking on a news article’s position in news aggregation rankings.

|  | | Fact-Checking | | | | | | | | | Fact-Checking and Voting | | | | | | | | |
| --- | --- | --- | --- | --- | --- | --- | --- | --- | --- | --- | --- | --- | --- | --- | --- | --- | --- | --- | --- |
| Model | Minutes | Coeﬀicient | stderr | 95% | Upper | ConfInt | 95% | Lower | ConfInt | p-value | Coeﬀicient | stderr | 95% | Upper | ConfInt | 95% | Lower | ConfInt | p-value |
| 1 | 4.00 | -7.74 | 6.68 | 8.22 | | | -23.70 | | | 0.74 | -3.70 | 6.66 | 12.22 | | | -19.61 | | | 1.00 |
| 2 | 8.00 | -6.19 | 6.99 | 10.52 | | | -22.91 | | | 1.00 | -3.30 | 6.98 | 13.39 | | | -19.99 | | | 1.00 |
| 3 | 12.00 | -11.61 | 7.41 | 6.11 | | | -29.33 | | | 0.35 | -5.03 | 7.41 | 12.69 | | | -22.75 | | | 1.00 |
| 4 | 16.00 | -11.19 | 7.47 | 6.66 | | | -29.03 | | | 0.40 | -6.27 | 7.46 | 11.56 | | | -24.10 | | | 1.00 |
| 5 | 20.00 | -13.59 | 7.64 | 4.66 | | | -31.84 | | | 0.23 | -7.82 | 7.62 | 10.38 | | | -26.03 | | | 0.91 |
| 6 | 24.00 | -14.34 | 7.87 | 4.48 | | | -33.16 | | | 0.21 | -6.31 | 7.86 | 12.47 | | | -25.09 | | | 1.00 |
| 7 | 28.00 | -19.74 | 7.93 | -0.78 | | | -38.69 | | | 0.04 | -7.73 | 7.92 | 11.20 | | | -26.67 | | | 0.99 |
| 8 | 32.00 | -18.05 | 7.92 | 0.89 | | | -36.98 | | | 0.07 | -8.08 | 7.91 | 10.83 | | | -27.00 | | | 0.92 |
| 9 | 36.00 | -21.13 | 8.05 | -1.88 | | | -40.38 | | | 0.03 | -10.97 | 8.05 | 8.26 | | | -30.20 | | | 0.52 |
| 10 | 40.00 | -20.31 | 8.17 | -0.77 | | | -39.85 | | | 0.04 | -10.19 | 8.16 | 9.30 | | | -29.69 | | | 0.64 |
| 11 | 44.00 | -22.08 | 8.27 | -2.30 | | | -41.85 | | | 0.02 | -9.50 | 8.27 | 10.26 | | | -29.26 | | | 0.75 |
| 12 | 48.00 | -21.19 | 8.23 | -1.52 | | | -40.85 | | | 0.03 | -9.18 | 8.23 | 10.48 | | | -28.85 | | | 0.79 |
| 13 | 52.00 | -21.14 | 8.20 | -1.54 | | | -40.75 | | | 0.03 | -7.00 | 8.20 | 12.60 | | | -26.60 | | | 1.00 |
| 14 | 56.00 | -23.11 | 8.20 | -3.51 | | | -42.72 | | | 0.02 | -6.66 | 8.19 | 12.91 | | | -26.23 | | | 1.00 |
| 15 | 60.00 | -23.65 | 8.24 | -3.95 | | | -43.35 | | | 0.01 | -7.41 | 8.22 | 12.24 | | | -27.05 | | | 1.00 |
| 16 | 64.00 | -24.67 | 8.19 | -5.11 | | | -44.23 | | | 0.01 | -8.73 | 8.16 | 10.76 | | | -28.23 | | | 0.85 |
| 17 | 68.00 | -23.79 | 8.20 | -4.18 | | | -43.39 | | | 0.01 | -7.95 | 8.18 | 11.61 | | | -27.51 | | | 1.00 |
| 18 | 72.00 | -23.82 | 8.23 | -4.14 | | | -43.50 | | | 0.01 | -9.89 | 8.22 | 9.76 | | | -29.53 | | | 0.69 |
| 19 | 76.00 | -21.84 | 8.26 | -2.10 | | | -41.58 | | | 0.03 | -9.62 | 8.25 | 10.09 | | | -29.34 | | | 0.73 |
| 20 | 80.00 | -21.64 | 8.25 | -1.91 | | | -41.36 | | | 0.03 | -8.80 | 8.25 | 10.91 | | | -28.50 | | | 0.86 |
| 21 | 84.00 | -21.78 | 8.26 | -2.04 | | | -41.52 | | | 0.03 | -9.39 | 8.25 | 10.33 | | | -29.11 | | | 0.77 |
| 22 | 88.00 | -20.41 | 8.22 | -0.77 | | | -40.06 | | | 0.04 | -8.79 | 8.21 | 10.83 | | | -28.40 | | | 0.85 |
| 23 | 92.00 | -18.60 | 8.26 | 1.13 | | | -38.33 | | | 0.07 | -6.94 | 8.25 | 12.78 | | | -26.65 | | | 1.00 |
| 24 | 96.00 | -19.38 | 8.27 | 0.38 | | | -39.14 | | | 0.06 | -7.60 | 8.25 | 12.12 | | | -27.33 | | | 1.00 |
| 25 | 100.00 | -19.84 | 8.29 | -0.03 | | | -39.65 | | | 0.05 | -7.94 | 8.28 | 11.85 | | | -27.74 | | | 1.00 |
| 26 | 104.00 | -20.56 | 8.34 | -0.62 | | | -40.49 | | | 0.04 | -8.67 | 8.33 | 11.24 | | | -28.59 | | | 0.90 |
| 27 | 108.00 | -19.85 | 8.33 | 0.05 | | | -39.76 | | | 0.05 | -8.85 | 8.32 | 11.05 | | | -28.74 | | | 0.86 |
| 28 | 112.00 | -19.08 | 8.35 | 0.87 | | | -39.04 | | | 0.07 | -8.09 | 8.35 | 11.86 | | | -28.04 | | | 1.00 |
| 29 | 116.00 | -19.82 | 8.35 | 0.13 | | | -39.77 | | | 0.05 | -7.39 | 8.34 | 12.55 | | | -27.33 | | | 1.00 |
| 30 | 120.00 | -18.15 | 8.27 | 1.62 | | | -37.92 | | | 0.09 | -6.86 | 8.27 | 12.91 | | | -26.63 | | | 1.00 |
| 31 | 124.00 | -18.22 | 8.32 | 1.66 | | | -38.11 | | | 0.09 | -6.98 | 8.31 | 12.88 | | | -26.84 | | | 1.00 |
| 32 | 128.00 | -19.20 | 8.30 | 0.63 | | | -39.03 | | | 0.06 | -6.83 | 8.27 | 12.94 | | | -26.60 | | | 1.00 |
| 33 | 132.00 | -17.62 | 8.26 | 2.12 | | | -37.36 | | | 0.10 | -6.02 | 8.25 | 13.70 | | | -25.74 | | | 1.00 |
| 34 | 136.00 | -18.10 | 8.26 | 1.65 | | | -37.84 | | | 0.09 | -6.42 | 8.26 | 13.32 | | | -26.16 | | | 1.00 |
| 35 | 140.00 | -19.16 | 8.23 | 0.51 | | | -38.82 | | | 0.06 | -6.66 | 8.23 | 13.02 | | | -26.34 | | | 1.00 |
| 36 | 144.00 | -18.51 | 8.20 | 1.08 | | | -38.10 | | | 0.07 | -6.44 | 8.21 | 13.18 | | | -26.05 | | | 1.00 |
| 37 | 148.00 | -18.66 | 8.20 | 0.94 | | | -38.26 | | | 0.07 | -6.64 | 8.21 | 12.98 | | | -26.26 | | | 1.00 |
| 38 | 152.00 | -18.61 | 8.17 | 0.92 | | | -38.15 | | | 0.07 | -6.62 | 8.18 | 12.92 | | | -26.17 | | | 1.00 |
| 39 | 156.00 | -18.45 | 8.18 | 1.10 | | | -38.00 | | | 0.07 | -6.84 | 8.17 | 12.69 | | | -26.37 | | | 1.00 |
| 40 | 160.00 | -17.95 | 8.19 | 1.62 | | | -37.51 | | | 0.09 | -6.36 | 8.18 | 13.19 | | | -25.92 | | | 1.00 |
| 41 | 164.00 | -17.53 | 8.20 | 2.06 | | | -37.13 | | | 0.10 | -6.31 | 8.19 | 13.27 | | | -25.90 | | | 1.00 |
| 42 | 168.00 | -17.20 | 8.21 | 2.42 | | | -36.81 | | | 0.11 | -5.88 | 8.20 | 13.73 | | | -25.48 | | | 1.00 |
| 43 | 172.00 | -17.06 | 8.18 | 2.48 | | | -36.60 | | | 0.11 | -5.35 | 8.18 | 14.19 | | | -24.89 | | | 1.00 |
| 44 | 176.00 | -17.67 | 8.20 | 1.92 | | | -37.26 | | | 0.09 | -5.51 | 8.19 | 14.06 | | | -25.07 | | | 1.00 |
| 45 | 180.00 | -17.87 | 8.18 | 1.69 | | | -37.42 | | | 0.09 | -6.21 | 8.17 | 13.33 | | | -25.74 | | | 1.00 |
| 46 | 184.00 | -18.36 | 8.17 | 1.18 | | | -37.89 | | | 0.08 | -6.05 | 8.16 | 13.46 | | | -25.57 | | | 1.00 |
| 47 | 188.00 | -17.55 | 8.14 | 1.92 | | | -37.01 | | | 0.09 | -5.90 | 8.14 | 13.56 | | | -25.35 | | | 1.00 |
| 48 | 192.00 | -17.52 | 8.15 | 1.97 | | | -37.00 | | | 0.10 | -6.30 | 8.15 | 13.18 | | | -25.77 | | | 1.00 |
| 49 | 196.00 | -17.02 | 8.14 | 2.43 | | | -36.46 | | | 0.11 | -6.19 | 8.14 | 13.27 | | | -25.64 | | | 1.00 |
| 50 | 200.00 | -17.84 | 8.11 | 1.55 | | | -37.22 | | | 0.08 | -6.90 | 8.12 | 12.50 | | | -26.29 | | | 1.00 |
| 51 | 204.00 | -18.16 | 8.10 | 1.19 | | | -37.51 | | | 0.08 | -6.80 | 8.10 | 12.55 | | | -26.16 | | | 1.00 |
| 52 | 208.00 | -18.76 | 8.03 | 0.44 | | | -37.95 | | | 0.06 | -6.86 | 8.04 | 12.34 | | | -26.07 | | | 1.00 |
| 53 | 212.00 | -17.83 | 8.03 | 1.36 | | | -37.03 | | | 0.08 | -6.25 | 8.04 | 12.96 | | | -25.47 | | | 1.00 |
| 54 | 216.00 | -19.15 | 8.00 | -0.03 | | | -38.26 | | | 0.05 | -6.43 | 8.00 | 12.69 | | | -25.55 | | | 1.00 |
| 55 | 220.00 | -18.47 | 8.01 | 0.67 | | | -37.61 | | | 0.06 | -6.56 | 8.00 | 12.57 | | | -25.69 | | | 1.00 |
| 56 | 224.00 | -18.81 | 8.01 | 0.33 | | | -37.94 | | | 0.06 | -6.32 | 8.00 | 12.81 | | | -25.44 | | | 1.00 |
| 57 | 228.00 | -17.67 | 8.03 | 1.52 | | | -36.87 | | | 0.08 | -4.97 | 8.03 | 14.21 | | | -24.15 | | | 1.00 |
| 58 | 232.00 | -18.84 | 8.01 | 0.30 | | | -37.97 | | | 0.06 | -6.17 | 8.01 | 12.97 | | | -25.30 | | | 1.00 |
| 59 | 236.00 | -18.77 | 8.00 | 0.35 | | | -37.88 | | | 0.06 | -6.53 | 7.98 | 12.55 | | | -25.60 | | | 1.00 |
| 60 | 240.00 | -19.02 | 7.97 | 0.03 | | | -38.07 | | | 0.05 | -6.79 | 7.97 | 12.25 | | | -25.83 | | | 1.00 |
| 61 | 244.00 | -19.01 | 7.97 | 0.04 | | | -38.06 | | | 0.05 | -6.61 | 7.97 | 12.43 | | | -25.65 | | | 1.00 |
| 62 | 248.00 | -18.87 | 7.95 | 0.12 | | | -37.87 | | | 0.05 | -7.46 | 7.95 | 11.54 | | | -26.46 | | | 1.00 |
| 63 | 252.00 | -19.06 | 7.97 | -0.01 | | | -38.10 | | | 0.05 | -7.66 | 7.97 | 11.39 | | | -26.70 | | | 1.00 |
| 64 | 256.00 | -18.93 | 7.98 | 0.16 | | | -38.01 | | | 0.05 | -7.35 | 7.98 | 11.72 | | | -26.43 | | | 1.00 |
| 65 | 260.00 | -18.07 | 7.98 | 1.00 | | | -37.15 | | | 0.07 | -7.35 | 7.97 | 11.70 | | | -26.40 | | | 1.00 |
| 66 | 264.00 | -17.17 | 8.00 | 1.96 | | | -36.30 | | | 0.10 | -7.14 | 8.00 | 11.97 | | | -26.25 | | | 1.00 |
| 67 | 268.00 | -17.33 | 8.02 | 1.84 | | | -36.50 | | | 0.09 | -7.00 | 8.01 | 12.15 | | | -26.16 | | | 1.00 |
| 68 | 272.00 | -17.82 | 7.99 | 1.27 | | | -36.92 | | | 0.08 | -6.71 | 7.98 | 12.37 | | | -25.79 | | | 1.00 |
| 69 | 276.00 | -16.72 | 7.93 | 2.24 | | | -35.69 | | | 0.11 | -5.62 | 7.93 | 13.34 | | | -24.58 | | | 1.00 |
| 70 | 280.00 | -16.55 | 7.94 | 2.44 | | | -35.54 | | | 0.11 | -5.85 | 7.94 | 13.14 | | | -24.83 | | | 1.00 |
| 71 | 284.00 | -16.72 | 7.97 | 2.32 | | | -35.76 | | | 0.11 | -5.78 | 7.95 | 13.23 | | | -24.79 | | | 1.00 |
| 72 | 288.00 | -16.26 | 7.92 | 2.69 | | | -35.20 | | | 0.12 | -6.92 | 7.90 | 11.97 | | | -25.81 | | | 1.00 |
| 73 | 292.00 | -15.13 | 7.90 | 3.76 | | | -34.02 | | | 0.17 | -6.17 | 7.88 | 12.66 | | | -25.01 | | | 1.00 |
| 74 | 296.00 | -14.62 | 7.90 | 4.27 | | | -33.50 | | | 0.19 | -6.02 | 7.89 | 12.84 | | | -24.88 | | | 1.00 |
| 75 | 300.00 | -14.57 | 7.90 | 4.30 | | | -33.45 | | | 0.20 | -5.74 | 7.88 | 13.10 | | | -24.58 | | | 1.00 |
| 76 | 304.00 | -14.96 | 7.89 | 3.89 | | | -33.82 | | | 0.18 | -5.87 | 7.88 | 12.96 | | | -24.70 | | | 1.00 |
| 77 | 308.00 | -14.57 | 7.89 | 4.29 | | | -33.44 | | | 0.20 | -5.78 | 7.90 | 13.09 | | | -24.65 | | | 1.00 |
| 78 | 312.00 | -14.48 | 7.89 | 4.38 | | | -33.33 | | | 0.20 | -5.96 | 7.90 | 12.91 | | | -24.84 | | | 1.00 |
| 79 | 316.00 | -14.97 | 7.91 | 3.94 | | | -33.89 | | | 0.18 | -6.09 | 7.92 | 12.83 | | | -25.02 | | | 1.00 |
| 80 | 320.00 | -15.64 | 7.83 | 3.07 | | | -34.34 | | | 0.14 | -8.14 | 7.83 | 10.57 | | | -26.86 | | | 0.90 |
| 81 | 324.00 | -14.76 | 7.80 | 3.89 | | | -33.41 | | | 0.18 | -6.74 | 7.81 | 11.91 | | | -25.40 | | | 1.00 |
| 82 | 328.00 | -16.25 | 7.82 | 2.44 | | | -34.93 | | | 0.11 | -8.01 | 7.82 | 10.68 | | | -26.70 | | | 0.92 |
| 83 | 332.00 | -15.51 | 7.82 | 3.17 | | | -34.20 | | | 0.14 | -7.46 | 7.82 | 11.23 | | | -26.14 | | | 1.00 |
| 84 | 336.00 | -15.65 | 7.83 | 3.07 | | | -34.36 | | | 0.14 | -7.62 | 7.83 | 11.09 | | | -26.33 | | | 0.99 |
| 85 | 340.00 | -15.29 | 7.84 | 3.46 | | | -34.03 | | | 0.16 | -7.54 | 7.84 | 11.20 | | | -26.29 | | | 1.00 |
| 86 | 344.00 | -15.42 | 7.84 | 3.32 | | | -34.17 | | | 0.15 | -7.86 | 7.85 | 10.90 | | | -26.62 | | | 0.95 |
| 87 | 348.00 | -15.87 | 7.83 | 2.84 | | | -34.59 | | | 0.13 | -8.22 | 7.84 | 10.51 | | | -26.95 | | | 0.88 |
| 88 | 352.00 | -15.26 | 7.81 | 3.41 | | | -33.92 | | | 0.15 | -7.94 | 7.82 | 10.76 | | | -26.64 | | | 0.93 |
| 89 | 356.00 | -14.99 | 7.79 | 3.63 | | | -33.60 | | | 0.16 | -8.73 | 7.81 | 9.93 | | | -27.40 | | | 0.79 |
| 90 | 360.00 | -15.69 | 7.79 | 2.93 | | | -34.31 | | | 0.13 | -8.77 | 7.81 | 9.90 | | | -27.43 | | | 0.79 |
| 91 | 364.00 | -15.73 | 7.79 | 2.90 | | | -34.36 | | | 0.13 | -9.18 | 7.81 | 9.50 | | | -27.85 | | | 0.72 |
| 92 | 368.00 | -15.35 | 7.79 | 3.28 | | | -33.98 | | | 0.15 | -9.19 | 7.81 | 9.47 | | | -27.86 | | | 0.72 |
| 93 | 372.00 | -14.96 | 7.78 | 3.63 | | | -33.54 | | | 0.17 | -8.99 | 7.79 | 9.63 | | | -27.62 | | | 0.75 |
| 94 | 376.00 | -14.68 | 7.78 | 3.92 | | | -33.28 | | | 0.18 | -9.08 | 7.79 | 9.53 | | | -27.69 | | | 0.73 |
| 95 | 380.00 | -14.44 | 7.76 | 4.11 | | | -32.99 | | | 0.19 | -9.41 | 7.78 | 9.18 | | | -27.99 | | | 0.68 |
| 96 | 384.00 | -13.21 | 7.79 | 5.42 | | | -31.83 | | | 0.27 | -8.29 | 7.81 | 10.36 | | | -26.95 | | | 0.87 |
| 97 | 388.00 | -13.22 | 7.79 | 5.39 | | | -31.83 | | | 0.27 | -8.38 | 7.80 | 10.28 | | | -27.03 | | | 0.85 |
| 98 | 392.00 | -12.81 | 7.78 | 5.79 | | | -31.40 | | | 0.30 | -8.27 | 7.80 | 10.37 | | | -26.91 | | | 0.87 |
| 99 | 396.00 | -12.47 | 7.77 | 6.10 | | | -31.04 | | | 0.33 | -7.98 | 7.80 | 10.66 | | | -26.62 | | | 0.92 |
| 100 | 400.00 | -12.81 | 7.78 | 5.78 | | | -31.40 | | | 0.30 | -8.13 | 7.81 | 10.53 | | | -26.78 | | | 0.90 |
| 101 | 404.00 | -12.65 | 7.80 | 5.99 | | | -31.29 | | | 0.32 | -8.43 | 7.81 | 10.23 | | | -27.08 | | | 0.84 |
| 102 | 408.00 | -12.70 | 7.79 | 5.92 | | | -31.32 | | | 0.31 | -8.30 | 7.80 | 10.35 | | | -26.96 | | | 0.86 |
| 103 | 412.00 | -11.25 | 7.74 | 7.25 | | | -29.76 | | | 0.44 | -7.14 | 7.77 | 11.42 | | | -25.71 | | | 1.00 |
| 104 | 416.00 | -11.17 | 7.76 | 7.38 | | | -29.72 | | | 0.45 | -6.93 | 7.78 | 11.65 | | | -25.52 | | | 1.00 |
| 105 | 420.00 | -10.95 | 7.73 | 7.54 | | | -29.43 | | | 0.47 | -6.86 | 7.76 | 11.69 | | | -25.42 | | | 1.00 |

Table S6: Average treatment effect coeﬀicients and test statistics from 105 linear regression models testing the average treatment effects on the rank position of a news article in the top 300 rank positions at a moment of time after the article was posted.

|  | | Fact-Checking | | | | | | | | | Fact-Checking and Voting | | | | | | | | |
| --- | --- | --- | --- | --- | --- | --- | --- | --- | --- | --- | --- | --- | --- | --- | --- | --- | --- | --- | --- |
| Model | Minutes | Coeﬀicient | stderr | 95% | Upper | ConfInt | 95% | Lower | ConfInt | p-value | Coeﬀicient | stderr | 95% | Upper | ConfInt | 95% | Lower | ConfInt | p-value |
| 1 | 4.00 | -0.22 | 0.16 | 0.10 | | | -0.54 | | | 0.18 | 0.01 | 0.16 | 0.33 | | | -0.31 | | | 0.94 |
| 2 | 8.00 | -0.17 | 0.21 | 0.24 | | | -0.58 | | | 0.41 | -0.12 | 0.21 | 0.29 | | | -0.53 | | | 0.57 |
| 3 | 12.00 | -0.34 | 0.26 | 0.17 | | | -0.86 | | | 0.19 | -0.20 | 0.26 | 0.31 | | | -0.71 | | | 0.45 |
| 4 | 16.00 | -0.49 | 0.31 | 0.13 | | | -1.10 | | | 0.12 | -0.33 | 0.31 | 0.29 | | | -0.94 | | | 0.30 |
| 5 | 20.00 | -0.70 | 0.38 | 0.03 | | | -1.44 | | | 0.06 | -0.64 | 0.38 | 0.09 | | | -1.38 | | | 0.09 |
| 6 | 24.00 | -0.99 | 0.45 | -0.09 | | | -1.88 | | | 0.03 | -0.91 | 0.45 | -0.02 | | | -1.80 | | | 0.04 |
| 7 | 28.00 | -1.22 | 0.52 | -0.20 | | | -2.24 | | | 0.02 | -1.08 | 0.52 | -0.06 | | | -2.10 | | | 0.04 |
| 8 | 32.00 | -1.57 | 0.58 | -0.43 | | | -2.71 | | | 0.01 | -1.37 | 0.58 | -0.24 | | | -2.51 | | | 0.02 |
| 9 | 36.00 | -1.78 | 0.65 | -0.50 | | | -3.05 | | | 0.01 | -1.50 | 0.65 | -0.23 | | | -2.77 | | | 0.02 |
| 10 | 40.00 | -2.07 | 0.74 | -0.63 | | | -3.51 | | | 0.01 | -1.79 | 0.73 | -0.35 | | | -3.22 | | | 0.02 |
| 11 | 44.00 | -2.33 | 0.81 | -0.73 | | | -3.92 | | | 0.00 | -1.86 | 0.81 | -0.27 | | | -3.45 | | | 0.02 |
| 12 | 48.00 | -2.50 | 0.88 | -0.78 | | | -4.22 | | | 0.00 | -2.09 | 0.88 | -0.37 | | | -3.81 | | | 0.02 |
| 13 | 52.00 | -2.70 | 0.96 | -0.82 | | | -4.58 | | | 0.01 | -2.11 | 0.96 | -0.23 | | | -3.99 | | | 0.03 |
| 14 | 56.00 | -2.93 | 1.07 | -0.84 | | | -5.02 | | | 0.01 | -2.34 | 1.07 | -0.25 | | | -4.42 | | | 0.03 |
| 15 | 60.00 | -3.17 | 1.14 | -0.94 | | | -5.40 | | | 0.01 | -2.41 | 1.13 | -0.18 | | | -4.63 | | | 0.03 |
| 16 | 64.00 | -3.42 | 1.24 | -0.99 | | | -5.86 | | | 0.01 | -2.58 | 1.24 | -0.16 | | | -5.01 | | | 0.04 |
| 17 | 68.00 | -3.76 | 1.33 | -1.15 | | | -6.37 | | | 0.00 | -2.72 | 1.33 | -0.11 | | | -5.33 | | | 0.04 |
| 18 | 72.00 | -4.04 | 1.41 | -1.27 | | | -6.81 | | | 0.00 | -3.03 | 1.41 | -0.26 | | | -5.79 | | | 0.03 |
| 19 | 76.00 | -4.28 | 1.50 | -1.33 | | | -7.23 | | | 0.00 | -3.36 | 1.50 | -0.41 | | | -6.31 | | | 0.03 |
| 20 | 80.00 | -4.46 | 1.58 | -1.36 | | | -7.57 | | | 0.01 | -3.42 | 1.58 | -0.32 | | | -6.53 | | | 0.03 |
| 21 | 84.00 | -5.03 | 1.69 | -1.72 | | | -8.34 | | | 0.00 | -4.05 | 1.69 | -0.74 | | | -7.36 | | | 0.02 |
| 22 | 88.00 | -5.23 | 1.82 | -1.65 | | | -8.80 | | | 0.00 | -4.14 | 1.82 | -0.57 | | | -7.71 | | | 0.02 |
| 23 | 92.00 | -5.46 | 1.93 | -1.68 | | | -9.24 | | | 0.00 | -4.51 | 1.93 | -0.73 | | | -8.29 | | | 0.02 |
| 24 | 96.00 | -5.83 | 2.03 | -1.84 | | | -9.81 | | | 0.00 | -4.63 | 2.03 | -0.66 | | | -8.61 | | | 0.02 |
| 25 | 100.00 | -6.25 | 2.15 | -2.05 | | | -10.46 | | | 0.00 | -5.04 | 2.14 | -0.84 | | | -9.24 | | | 0.02 |
| 26 | 104.00 | -6.43 | 2.27 | -1.99 | | | -10.88 | | | 0.00 | -5.18 | 2.26 | -0.75 | | | -9.62 | | | 0.02 |
| 27 | 108.00 | -6.92 | 2.39 | -2.23 | | | -11.61 | | | 0.00 | -5.61 | 2.39 | -0.91 | | | -10.30 | | | 0.02 |
| 28 | 112.00 | -7.35 | 2.54 | -2.38 | | | -12.32 | | | 0.00 | -6.02 | 2.53 | -1.05 | | | -10.99 | | | 0.02 |
| 29 | 116.00 | -7.73 | 2.67 | -2.49 | | | -12.97 | | | 0.00 | -6.53 | 2.67 | -1.29 | | | -11.76 | | | 0.01 |
| 30 | 120.00 | -8.38 | 2.82 | -2.86 | | | -13.91 | | | 0.00 | -6.91 | 2.82 | -1.39 | | | -12.44 | | | 0.01 |
| 31 | 124.00 | -8.63 | 2.96 | -2.83 | | | -14.44 | | | 0.00 | -7.27 | 2.96 | -1.47 | | | -13.07 | | | 0.01 |
| 32 | 128.00 | -9.04 | 3.08 | -3.01 | | | -15.08 | | | 0.00 | -7.67 | 3.07 | -1.66 | | | -13.69 | | | 0.01 |
| 33 | 132.00 | -9.38 | 3.21 | -3.08 | | | -15.68 | | | 0.00 | -8.00 | 3.21 | -1.71 | | | -14.29 | | | 0.01 |
| 34 | 136.00 | -10.11 | 3.38 | -3.49 | | | -16.74 | | | 0.00 | -8.90 | 3.38 | -2.28 | | | -15.52 | | | 0.01 |
| 35 | 140.00 | -10.40 | 3.55 | -3.44 | | | -17.37 | | | 0.00 | -9.32 | 3.56 | -2.35 | | | -16.29 | | | 0.01 |
| 36 | 144.00 | -11.01 | 3.72 | -3.72 | | | -18.29 | | | 0.00 | -9.79 | 3.72 | -2.50 | | | -17.09 | | | 0.01 |
| 37 | 148.00 | -11.67 | 3.96 | -3.92 | | | -19.43 | | | 0.00 | -10.35 | 3.96 | -2.58 | | | -18.12 | | | 0.01 |
| 38 | 152.00 | -12.19 | 4.20 | -3.97 | | | -20.41 | | | 0.00 | -11.27 | 4.20 | -3.05 | | | -19.50 | | | 0.01 |
| 39 | 156.00 | -12.94 | 4.43 | -4.25 | | | -21.62 | | | 0.00 | -11.79 | 4.43 | -3.11 | | | -20.46 | | | 0.01 |
| 40 | 160.00 | -13.65 | 4.70 | -4.43 | | | -22.87 | | | 0.00 | -12.63 | 4.70 | -3.42 | | | -21.84 | | | 0.01 |
| 41 | 164.00 | -14.16 | 5.03 | -4.31 | | | -24.01 | | | 0.01 | -13.32 | 5.02 | -3.47 | | | -23.16 | | | 0.01 |
| 42 | 168.00 | -14.84 | 5.33 | -4.40 | | | -25.28 | | | 0.01 | -13.86 | 5.33 | -3.42 | | | -24.29 | | | 0.01 |
| 43 | 172.00 | -16.31 | 5.96 | -4.62 | | | -28.00 | | | 0.01 | -15.49 | 5.97 | -3.80 | | | -27.18 | | | 0.01 |
| 44 | 176.00 | -18.15 | 6.80 | -4.81 | | | -31.49 | | | 0.01 | -17.15 | 6.80 | -3.83 | | | -30.48 | | | 0.01 |
| 45 | 180.00 | -19.98 | 7.92 | -4.46 | | | -35.50 | | | 0.01 | -18.68 | 7.91 | -3.18 | | | -34.19 | | | 0.02 |
| 46 | 184.00 | -22.36 | 9.19 | -4.34 | | | -40.38 | | | 0.02 | -21.01 | 9.18 | -3.01 | | | -39.01 | | | 0.02 |
| 47 | 188.00 | -25.49 | 11.07 | -3.78 | | | -47.19 | | | 0.02 | -24.07 | 11.07 | -2.37 | | | -45.77 | | | 0.03 |
| 48 | 192.00 | -28.02 | 12.99 | -2.55 | | | -53.48 | | | 0.03 | -26.88 | 12.99 | -1.43 | | | -52.34 | | | 0.04 |
| 49 | 196.00 | -30.88 | 14.92 | -1.64 | | | -60.12 | | | 0.04 | -29.66 | 14.92 | -0.41 | | | -58.92 | | | 0.05 |
| 50 | 200.00 | -34.48 | 17.28 | -0.61 | | | -68.35 | | | 0.05 | -33.36 | 17.29 | 0.53 | | | -67.25 | | | 0.05 |
| 51 | 204.00 | -37.42 | 19.53 | 0.86 | | | -75.71 | | | 0.06 | -36.24 | 19.54 | 2.06 | | | -74.53 | | | 0.06 |
| 52 | 208.00 | -40.74 | 21.82 | 2.03 | | | -83.50 | | | 0.06 | -39.34 | 21.83 | 3.45 | | | -82.12 | | | 0.07 |
| 53 | 212.00 | -44.27 | 24.30 | 3.36 | | | -91.89 | | | 0.07 | -42.87 | 24.33 | 4.80 | | | -90.55 | | | 0.08 |
| 54 | 216.00 | -48.17 | 27.11 | 4.97 | | | -101.30 | | | 0.08 | -46.42 | 27.12 | 6.73 | | | -99.58 | | | 0.09 |
| 55 | 220.00 | -51.67 | 29.73 | 6.61 | | | -109.95 | | | 0.08 | -50.19 | 29.72 | 8.06 | | | -108.45 | | | 0.09 |
| 56 | 224.00 | -55.58 | 32.57 | 8.26 | | | -119.42 | | | 0.09 | -53.65 | 32.56 | 10.16 | | | -117.47 | | | 0.10 |
| 57 | 228.00 | -59.47 | 35.14 | 9.40 | | | -128.34 | | | 0.09 | -57.37 | 35.12 | 11.46 | | | -126.20 | | | 0.10 |
| 58 | 232.00 | -64.43 | 38.60 | 11.22 | | | -140.08 | | | 0.10 | -62.16 | 38.60 | 13.49 | | | -137.81 | | | 0.11 |
| 59 | 236.00 | -68.03 | 41.60 | 13.51 | | | -149.58 | | | 0.10 | -65.45 | 41.51 | 15.91 | | | -146.81 | | | 0.12 |
| 60 | 240.00 | -72.88 | 44.65 | 14.64 | | | -160.41 | | | 0.10 | -69.92 | 44.63 | 17.55 | | | -157.39 | | | 0.12 |
| 61 | 244.00 | -77.28 | 47.92 | 16.65 | | | -171.21 | | | 0.11 | -74.23 | 47.89 | 19.64 | | | -168.10 | | | 0.12 |
| 62 | 248.00 | -82.18 | 51.61 | 18.97 | | | -183.33 | | | 0.11 | -78.82 | 51.61 | 22.34 | | | -179.97 | | | 0.13 |
| 63 | 252.00 | -86.84 | 54.86 | 20.69 | | | -194.37 | | | 0.11 | -83.59 | 54.85 | 23.93 | | | -191.10 | | | 0.13 |
| 64 | 256.00 | -91.32 | 57.88 | 22.13 | | | -204.78 | | | 0.12 | -87.49 | 57.86 | 25.92 | | | -200.90 | | | 0.13 |
| 65 | 260.00 | -95.80 | 61.00 | 23.76 | | | -215.36 | | | 0.12 | -91.83 | 60.92 | 27.57 | | | -211.24 | | | 0.13 |
| 66 | 264.00 | -99.64 | 63.85 | 25.51 | | | -224.78 | | | 0.12 | -96.02 | 63.79 | 29.00 | | | -221.04 | | | 0.13 |
| 67 | 268.00 | -103.16 | 66.29 | 26.78 | | | -233.09 | | | 0.12 | -99.39 | 66.24 | 30.45 | | | -229.23 | | | 0.13 |
| 68 | 272.00 | -107.55 | 69.16 | 27.99 | | | -243.10 | | | 0.12 | -103.24 | 69.11 | 32.20 | | | -238.69 | | | 0.14 |
| 69 | 276.00 | -109.98 | 71.68 | 30.52 | | | -250.48 | | | 0.13 | -105.96 | 71.67 | 34.51 | | | -246.43 | | | 0.14 |
| 70 | 280.00 | -113.80 | 74.37 | 31.96 | | | -259.56 | | | 0.13 | -109.56 | 74.36 | 36.18 | | | -255.30 | | | 0.14 |
| 71 | 284.00 | -118.57 | 77.46 | 33.25 | | | -270.39 | | | 0.13 | -114.18 | 77.34 | 37.42 | | | -265.77 | | | 0.14 |
| 72 | 288.00 | -122.71 | 79.83 | 33.76 | | | -279.17 | | | 0.12 | -117.73 | 79.62 | 38.34 | | | -273.79 | | | 0.14 |
| 73 | 292.00 | -127.15 | 82.78 | 35.10 | | | -289.40 | | | 0.13 | -122.12 | 82.57 | 39.71 | | | -283.95 | | | 0.14 |
| 74 | 296.00 | -130.61 | 85.25 | 36.48 | | | -297.70 | | | 0.13 | -125.67 | 85.14 | 41.21 | | | -292.55 | | | 0.14 |
| 75 | 300.00 | -134.34 | 87.96 | 38.05 | | | -306.74 | | | 0.13 | -129.03 | 87.80 | 43.06 | | | -301.12 | | | 0.14 |
| 76 | 304.00 | -138.26 | 90.47 | 39.07 | | | -315.58 | | | 0.13 | -133.35 | 90.38 | 43.79 | | | -310.49 | | | 0.14 |
| 77 | 308.00 | -140.99 | 92.56 | 40.44 | | | -322.41 | | | 0.13 | -136.23 | 92.58 | 45.22 | | | -317.69 | | | 0.14 |
| 78 | 312.00 | -144.48 | 94.86 | 41.45 | | | -330.40 | | | 0.13 | -139.83 | 94.95 | 46.26 | | | -325.93 | | | 0.14 |
| 79 | 316.00 | -147.98 | 97.10 | 42.34 | | | -338.29 | | | 0.13 | -143.84 | 97.13 | 46.54 | | | -334.23 | | | 0.14 |
| 80 | 320.00 | -151.24 | 99.12 | 43.04 | | | -345.51 | | | 0.13 | -146.20 | 99.18 | 48.19 | | | -340.60 | | | 0.14 |
| 81 | 324.00 | -155.01 | 101.23 | 43.41 | | | -353.42 | | | 0.13 | -150.29 | 101.28 | 48.21 | | | -348.80 | | | 0.14 |
| 82 | 328.00 | -160.00 | 103.56 | 42.97 | | | -362.97 | | | 0.12 | -154.97 | 103.57 | 48.03 | | | -357.97 | | | 0.14 |
| 83 | 332.00 | -162.26 | 105.32 | 44.16 | | | -368.69 | | | 0.12 | -157.38 | 105.33 | 49.07 | | | -363.82 | | | 0.14 |
| 84 | 336.00 | -166.11 | 107.24 | 44.09 | | | -376.31 | | | 0.12 | -160.93 | 107.21 | 49.19 | | | -371.05 | | | 0.13 |
| 85 | 340.00 | -169.86 | 109.35 | 44.47 | | | -384.20 | | | 0.12 | -165.01 | 109.36 | 49.34 | | | -379.36 | | | 0.13 |
| 86 | 344.00 | -174.51 | 111.67 | 44.36 | | | -393.38 | | | 0.12 | -170.30 | 111.77 | 48.77 | | | -389.38 | | | 0.13 |
| 87 | 348.00 | -179.55 | 113.75 | 43.40 | | | -402.50 | | | 0.12 | -175.02 | 113.83 | 48.09 | | | -398.12 | | | 0.12 |
| 88 | 352.00 | -184.42 | 115.90 | 42.74 | | | -411.58 | | | 0.11 | -180.00 | 116.11 | 47.57 | | | -407.57 | | | 0.12 |
| 89 | 356.00 | -188.59 | 117.81 | 42.32 | | | -419.49 | | | 0.11 | -184.16 | 118.15 | 47.42 | | | -415.74 | | | 0.12 |
| 90 | 360.00 | -193.34 | 119.86 | 41.59 | | | -428.27 | | | 0.11 | -187.98 | 120.13 | 47.47 | | | -423.44 | | | 0.12 |
| 91 | 364.00 | -197.76 | 121.87 | 41.11 | | | -436.62 | | | 0.11 | -192.71 | 122.16 | 46.72 | | | -432.13 | | | 0.12 |
| 92 | 368.00 | -201.26 | 123.74 | 41.27 | | | -443.79 | | | 0.10 | -196.43 | 123.98 | 46.57 | | | -439.44 | | | 0.11 |
| 93 | 372.00 | -205.20 | 125.35 | 40.49 | | | -450.89 | | | 0.10 | -199.75 | 125.60 | 46.42 | | | -445.93 | | | 0.11 |
| 94 | 376.00 | -210.55 | 127.20 | 38.77 | | | -459.87 | | | 0.10 | -203.85 | 127.26 | 45.58 | | | -453.29 | | | 0.11 |
| 95 | 380.00 | -214.80 | 128.92 | 37.89 | | | -467.48 | | | 0.10 | -209.43 | 129.14 | 43.68 | | | -462.54 | | | 0.11 |
| 96 | 384.00 | -219.04 | 130.72 | 37.17 | | | -475.25 | | | 0.09 | -213.25 | 130.93 | 43.38 | | | -469.88 | | | 0.10 |
| 97 | 388.00 | -222.84 | 132.52 | 36.89 | | | -482.57 | | | 0.09 | -218.01 | 132.82 | 42.33 | | | -478.34 | | | 0.10 |
| 98 | 392.00 | -225.37 | 133.87 | 37.02 | | | -487.75 | | | 0.09 | -220.64 | 134.17 | 42.34 | | | -483.62 | | | 0.10 |
| 99 | 396.00 | -228.28 | 135.59 | 37.47 | | | -494.02 | | | 0.09 | -224.86 | 136.10 | 41.89 | | | -491.62 | | | 0.10 |
| 100 | 400.00 | -232.95 | 137.31 | 36.18 | | | -502.09 | | | 0.09 | -229.19 | 137.80 | 40.90 | | | -499.28 | | | 0.10 |
| 101 | 404.00 | -236.27 | 139.50 | 37.14 | | | -509.68 | | | 0.09 | -233.71 | 139.61 | 39.92 | | | -507.35 | | | 0.09 |
| 102 | 408.00 | -240.36 | 141.03 | 36.05 | | | -516.77 | | | 0.09 | -237.71 | 141.27 | 39.18 | | | -514.61 | | | 0.09 |
| 103 | 412.00 | -247.28 | 142.35 | 31.72 | | | -526.27 | | | 0.08 | -255.44 | 142.76 | 24.38 | | | -535.26 | | | 0.07 |
| 104 | 416.00 | -252.68 | 144.21 | 29.98 | | | -535.33 | | | 0.08 | -259.65 | 144.50 | 23.57 | | | -542.86 | | | 0.07 |
| 105 | 420.00 | -257.11 | 145.76 | 28.59 | | | -542.81 | | | 0.08 | -263.44 | 146.34 | 23.38 | | | -550.27 | | | 0.07 |

Table S7: Average treatment effect coeﬀicients and test statistics from 105 linear regression models testing the average treatment effects on the vote score of a news article in the top 300 rank positions at a moment of time after the article was posted.
